# Cortisol Levels in the Peri-implant Sulcular Fluid of Type-2 Diabetic and Non-diabetic Patients with Peri-implantitis

**DOI:** 10.3290/j.ohpd.b2960827

**Published:** 2022-04-27

**Authors:** Dena Ali, Jagan Kumar Baskaradoss, Bobby Karingada Joseph

**Affiliations:** a Associate Professor, Department of General Dental Practice, Kuwait University, Kuwait. Designed and supervised the study, acquired funding, wrote the manuscript and revised it prior to submission, measured the PISF volumes.; b Assistant Professor, Department of Developmental and Preventive Sciences, Kuwait University, Kuwait City, Kuwait. Wrote the manuscript and revised it prior to submission, performed the clinical investigations.; c Associate Professor, Department of Diagnostic Sciences, Faculty of Dentistry, Kuwait University, Safat, Kuwait. Wrote the manuscript and revised it prior to submission, performed the statistical analyses, assessed CL.

**Keywords:** cortisol, dental implant, inflammation, peri-implantitis, unstimulated whole saliva

## Abstract

**Purpose:**

Cortisol levels (CL) in peri-implant sulcular fluid (PISF) samples in relation to type-2 diabetes mellitus (T2DM) and peri-implantitis remain unaddressed. It is hypothesised that PISF CL are higher in patients with type-2 diabetes and peri-implantitis than in healthy patients without and with peri-implantitis. The aim was to assess the PISF CL of peri-implantitis patients without and with T2DM.

**Materials and Methods:**

Peri-implantitis patients with T2DM (group 1), T2DM patients without peri-implantitis (group 2), non-diabetic patients with peri-implantitis (group 3) and non-diabetic patients without peri-implantitis (group 4) were included. Demographics were recorded; and patients’ medical and dental records were assessed. Peri-implant modified plaque-index (mPI), modified gingival index (mGI), and probing depth (PD) and crestal bone loss (CBL) were recorded. The PISF was collected and CL were determined. p < 0.01 was considered statistically significant.

**Results:**

Each of the four groups included 16 subjects (n = 64) with no difference in mean age. In groups 1 and 2, the mean duration of T2DM was 10.5 ± 0.8 and 10.6 ± 0.4 years, respectively. Mean HbA1c levels (p < 0.01) were higher and clinicoradiographic parameters (p < 0.001) were worse in group 1 than in the other groups. The median PISF volume and mean CL were higher in groups 1 (p < 0.01) and 3 (p < 0.01) than groups 2 and 4. There was a statistically significant correlation between PD and CL in group 3 (p < 0.001).

**Conclusion:**

Cortisol levels in the PISF are higher in T2DM and non-diabetic patients with peri-implantitis than in healthy individuals with and without peri-implantitis. Hyperglycemia did not influence peri-implant clinicoradiographic parameters and CL in the present patient population.

Cortisol is a naturally occurring steroid hormone produced by the adrenal glands. It has been reported that under conditions of psychological stress, serum and salivary cortisol levels (CL) are elevated.^14,23,33^ From a dental perspective, it has been reported that cortisol is expressed in higher concentrations in patients with than without periodontitis.^2,19^ However, with reference to peri-implant diseases, to date there is only one study^4^ in the indexed literature that has assessed peri-implantitis with relation to CL in the peri-implant-sulcular-fluid (PISF). In a power-adjusted case-control study, Al-Resayes et al^4^ compared PISF-CL obtained from patients with peri-implantitis and controls. In that study, corticol levels in the PISF were measured using commercially available kits via enzyme-linked immunosorbent assay (ELISA). The results showed that PISF CLs were higher in patients with than without peri-implantitis. However, one limitation of the study by Alresayes et al^4^ was that patients with immunosuppressed health status were excluded.

Hyperglycemia is a hematological characteristic of poor controlled type-2 diabetes (T2DM);^39,40^ it is also an established risk factor for peri-implant mucositis and peri-implantitis.^17,26,40^ From an immunoinflammatory point of view, both peri-implantitis and T2DM are associated with increased production of destructive inflammatory cytokines, such as interleukin 1-beta (IL-1β), IL-6, and tumor necrosis factor alpha, which are expressed in high concentrations in the PISF of peri-implantitis patients.^1,15,38^ Moreover, chronic hyperglycemia induces oxidative stress (OS) in oral and systemic tissues and increases the expression of advanced glycation endproducts (AGEs) in the PISF, which in turn accelerates the overall inflammatory response. These immunoinflammatory mechanisms have also been linked to the etiopathogenesis of peri-implant diseases.^12^ No studies so far have correlated the expression and concentration of cortisol with glycemic levels among peri-implantitis patients with T2DM. It is hypothesised that PISF-CLs are higher in T2DM patients with peri-implantitis than among healthy controls with and without peri-implantitis.

The aim was to assess the PISF CLs of peri-implantitis patients without and with T2DM.

## Methods

### Ethics Statement

All procedures in the present investigation that involved human participants were performed in accordance with the ethical standards and approved by the ethics committee of the of the Health Science Center (HSC), Kuwait University (VDR/EC/3762; Dated: June 30, 2021). The study was performed in compliance with the 1964 Helsinki declaration and its later amendments or comparable ethical standards. Participation was voluntary and individuals were allowed to decline or withdraw their participation at any stage of investigation. Signing the consent form was mandatory for those who agreed to volunteer. All participants were informed about the objectives and methodology and were also allowed to ask questions.

### Participants and Eligibility Criteria

Self-reported T2DM and healthy individuals who had undergone dental implant treatment were included. Self-reported current nicotinic product users (including electronic cigarettes), habitual alcohol users, nursing or pregnant females and patients with systemic diseases other than T2DM were excluded. Furthermore, patients with a history of periodontitis, those who had undergone periodontal maintenance, and/or had used pharmacologic prescriptions of antibiotics, non-steroidal anti-inflammatory drugs and/or steroids in the past three months were not eligible.

### Questionnaire

A questionnaire was used to gather information related to the patient’s age, gender, family history and duration of T2DM, and daily toothbrushing and flossing. Individuals were also asked if they were aware of having any psychological conditions such as anxiety and depression and were seeking any treatment in this regard.

### Hemoglobin A1c

The HbA1c levels were evaluated by a trained and calibrated investigator (Kappa score 0.86) before clinicoradiographic examinations. The HbA1c levels were recorded using a digital device (QuoTest EKF Diagnostics; Magdeburg, Germany).

### Peri-implant Inflammatory Parameters

Peri-implant clinical (mPI, mGI and PD) and radiographic (CBL) measurements were carried out by one calibrated examiner (Kappa 0.86). The PI24, mGI27, and PD7 was assessed at 6 sites around implants using a graded plastic probe (Hu-Friedy; Chicago, IL, USA). Bitewing radiographs (Rinn XCP film holders, Dentsply; Elgin, IL, USA) were taken using E-speed films (Eastman Kodak; Rochester, NY, USA) and viewed using Planmeca Romexis software (Planmeca; Helsinki, Finland).

### Collection of Peri-implant Sulcular Fluid and Assessment of Cortisol Levels

PISF samples were performed 48 h after clinical and radiographic assessments. Collection of PISF samples was performed as described in a previous investigation.^4^ Peri-implant sites were isolated using sterile cotton rolls, and supra- and subgingival plaque was gently removed using plastic curettes (Hu-Friedy). Sterile paper strips (Periopaper, Interstate-Drug-Exchange; Amityville, NY, USA) were inserted in the mid-buccal peri-implant sulcus, held in place for 0.5 min, and then immediately assessed for PISF volume. The PISF volume was measured (Periotron 8000, OraFlow; Amityville, NY, USA) and strips were then placed in sterile plastic tubes (with lids) containing 1 ml buffered phosphate saline. The samples were kept at -82°C until further assessment. Strips contaminated with blood and saliva were discarded, and sampling was repeated after 1 h. All samples were assessed for CL within 48 h of collection.

### Cortisol Levels in the Peri-implant Sulcular Fluid

The CL were determined using an ELISA kit (EnzoCortisol ELISA/ADI/900/071, Farmingdale, NY, USA) according to the manufacturers’ instructions. The protocol for CL assessment is described elsewhere.^4^ The minimum detection limit was 56.7 pg/ml. Samples were then eluted with 500 μl PBS (pH 7.4). Samples (100 μl) and standards (100 μl) were added in duplicates to respective wells. An ELISA reader (Molecular-dynamics; Sunnyvale, CA, USA) was used at 450 nm to read light absorbance.

### Statistical and Power Analyses

Statistical comparisons (SPSS Version 26; Chicago, IL, USA) were performed via one-way ANOVA and Bonferroni post-hoc tests. The Kolmogorov-Smirnov test was used to assess data normality. Correlation of CL with severity of peri-implantitis was assessed using logistic regression analysis. p < 0.01 was deemed statistically significant. Power and sample sizes were determined using data from a pilot investigation (nQuery Advisor 6.0, StatisticalSolutions; Saugas, MA, USA) with an alpha and effect size of 1% and 0.3, respectively. With inclusion of at least 15 individuals per group (assuming a standard deviation of 1.0%), the study power was projected to be 83.4%.

## Results

### General Characteristics

Each of the four groups included 16 subjects (n = 64). Mean HbA1c levels were higher in group 1 than in groups 2 (p < 0.01), 3 (p < 0.01) and 4 (p < 0.01). A family history of DM was reported by 68.8% of the patients in group 1, 43.8% in group 2, 25% in groups 3 and 18.8% patients in group 4. Toothbrushing twice daily was reported by 75% of the patients in groups 2 and 87.5% in group 4 compared with 37.5% patients in groups 1 (37.5%) and 31.2% in group 3. Five patients in group 4 reported that they used dental floss once per day. These results are summarised in Table 1. Diagnosis and current/previous treatment for psychological disorders was not reported by any of the participants.

**Table 1:** Characteristics of the patient groups

Parameters	Group 1	Group 2	Group 3	Group 4
Patients (n)	16	16	16	16
Mean age in years	53.8 ± 5.6	52.5 ± 3.2	52.7 ± 1.6	52.2 ± 1.2
Duration of type-2 DM in years	10.5 ± 0.8	10.6 ± 0.4	NA	NA
HbA1c levels	9.5 ± 0.5*	4.5 ± 0.3	4.4 ± 0.2	4.3 ± 0.08
Family history of DM	11 (68.8%)	7 (43.8%)	4 (25%)	3 (18.8%)
Daily toothbrushing				
Once	10 (62.5%)	4 (25%)	11 (68.8%)	2 (12.5%)
Twice	6 (37.5%)	12 (75%)	5 (31.2%)	14 (87.5%)
Flossing				
Once	None	None	None	5 (31.3%)
Twice	None	None	None	None

Group 1: type-2 diabetic patients with peri-implantitis; group 2: type-2 diabetic patients without peri-implantitis; group 3: non-diabetic patients with peri-implantitis; group 4: non-diabetic patients without peri-implantitis. *Compared with group 2 (p < 0.01), 3 (p < 0.01) and 4 (p < 0.01). DM: diabetes mellitus.

### Characteristics of Dental Implants

**Fig 1:**
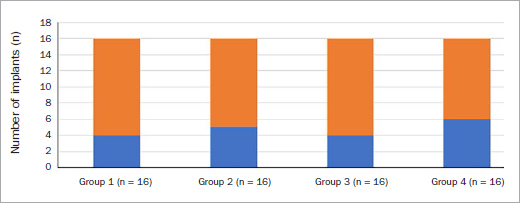
Implants located in the maxilla (blue) and mandible (orange) in groups 1 to 4.

There was no statistically significant difference in the mean duration of implants in function in all groups. All implants were delayed-loaded, platform-switched and placed using insertion torques ranging from 30 Ncm to 35 Ncm. The diameters and lengths of implants ranged from 4–4.1 mm and 11–13 mm, respectively. All implants were placed at bone level and had cement-retained restorations. Implant positioning in relation to jaw location is shown in Fig1.

### Clinicoradiographic Status

The mPI (p < 0.01), mGI (p < 0.01), PD (p < 0.01) and mesial (p < 0.01) and distal (p < 0.01) CBL were statistically significantly higher among patients in groups 1 and 3 than patients in groups 2 and 4 (Table 2).

**Table 2:** Peri-implant clinicoradiographic status in all groups

Parameters	Group 1 (n = 16)	Group 2 (n = 16)	Group 3 (n = 16)	Group 4 (n = 16)
Modified plaque index	3.1 ± 0.2*	0.8 ± 0.008	2.7 ± 0.06†	0.4 ± 0.005
Modified gingival index	3.3 ± 0.1*	0.3 ± 0.06	3.05 ± 0.05†	0.2 ± 0.005
Probing depth (in mm)	5.7 ± 0.4 mm*	0.9 ± 0.04 mm	5.2 ± 0.3 mm†	0.8 ± 0.05 mm
Crestal bone loss (mesial)	4.8 ± 0.2 mm*	0.7 ± 0.02 mm	4.5 ± 0.2 mm†	0.5 ± 0.01 mm
Crestal bone loss (distal)	4.6 ± 0.3 mm*	0.5 ± 0.003 mm	4.7 ± 0.1 mm†	0.5 ± 0.009 mm

Group 1: type-2 diabetic patients with peri-implantitis; group 2: type-2 diabetic patients without peri-implantitis; group 3: non-diabetic patients with peri-implantitis; group 4: non-diabetic patients without peri-implantitis. *Compared with groups 2 (p < 0.001) and 4 (p < 0.001). †Compared with groups 2 (p < 0.001) and 4 (p < 0.001).

### Volume of Peri-implant Sulcular Fluid and Cortisol Levels

**Fig 2:**
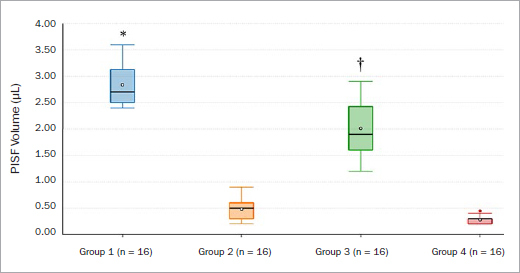
Median peri-implant sulcular fluid volume among patients in groups 1 to 4. *Comparison with groups 2 (p < 0.001) and 4 (p < 0.001). †Comparison with groups 2 (p < 0.01) and 4 (p < 0.01).

**Fig 3:**
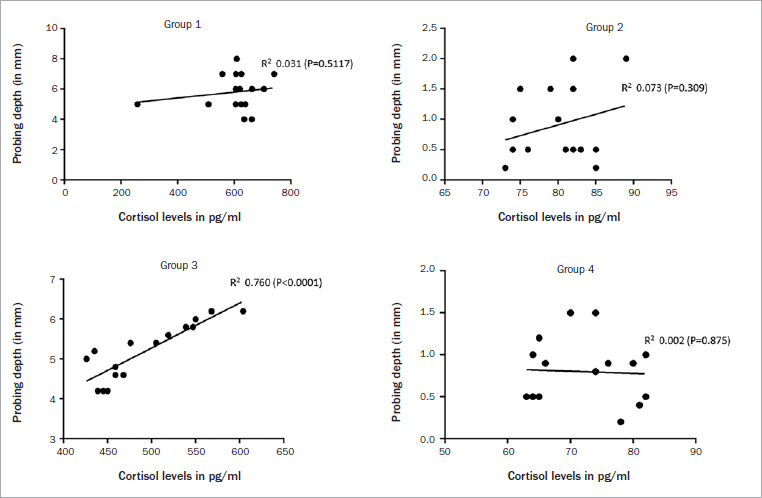
Correlation of peri-implant probing depth with cortisol levels among patients in groups 1 to 4.

The median PISF volume was higher in groups 1 (p < 0.001) and 3 (p < 0.01) than in the other groups (Fig 2). The mean PISF CLs were higher in group 1 (p < 0.001) than groups 2 and 4. The mean PISF CLs were statistically significantly higher among patients in group 3 (p < 0.001) compared with individuals in groups 2 and 4. There was no statistically significant difference in the mean PISF CLs among patients in groups 1 and 3 or groups 2 and 4 (Table 3). There was a statistically significant correlation between PISF CL and PD in group 3 (Table 4 and Fig 3). There was no statistically significant association between PISF volume and CL in relation to mPI, mGI, mesial and distal CBL, jaw location of implant, gender or HbA1c levels (data not shown).

**Table 3:** Mean (± SD) cortisol levels (pg/ml) in the peri-implant sulcular fluid of patients in all groups

Parameters	Group 1 (n = 16)	Group 2 (n = 16)	Group 3 (n = 16)	Group 4 (n = 16)
Cortisol levels	604.06 ± 87.69 pg/ml*	89.25 ± 10.04 pg/ml	538.77 ± 99.52 pg/ml†	73.08 ± 8.53 pg/ml

Group 1: type-2 diabetic patients with peri-implantitis; group 2: type-2 diabetic patients without peri-implantitis; group 3: non-diabetic patients with peri-implantitis; group 4: non-diabetic patients without peri-implantitis. *Compared with groups 2 (p < 0.001) and 4 (p < 0.001). †Compared with groups 2 (p < 0.001) and 4 (p < 0.001).

**Table 4:** Correlation of peri-implant probing depth with cortisol levels

Parameters	Slope	95% confidence interval	R2	F	DFn, DFd	p-value
group 1 (n = 16)	0.0019 ± 0.0028	-0.0042 to 0.0081	0.031	0.453	1, 14	0.5117
group 2 (n = 16)	0.0355 ± 0.0335	-0.0365 to 0.1072	0.073	1.112	1, 15	0.3098
group 3 (n = 16)	0.0112 ± 0.0016	0.00763 to 0.0148	0.7605	44.46	1, 14	<0.0001*
group 4 (n = 16)	-0.0025 ± 0.0137	-0.0321 to 0.0270	0.0023	0.033	1, 14	0.8573

## Discussion

The present study tested the hypothesis that CL in the PISF are higher among T2DM patients with peri-implantitis than in systemically healthy individuals with and without peri-implantitis. In other words, it was speculated that the results would demonstrate statistically significantly elevated PISF CLs in T2DM patients. However, this was not the case when the clinicoradiographic and immunoinflammatory parameters were statistically evaluated. Following the Bonferroni post-hoc adjustments, there was no statistically significant difference in the mean PISL CL among patients in groups 2 and 4. Moreover, another perplexing outcome was that the PISF CLs were comparable between T2DM and non-diabetic patients with peri-implantitis (groups 1 and 3, respectively). One clarification for this may be related to the results obtained from clinicoradiographic and hematologic investigations. It is worth mentioning that there was no difference in the clinicoradiographic parameters among T2DM and non-diabetic patients with periodontitis. Moreover, the mean HbA1c levels were also statistically non-significant among patients in groups 1 and 3, that is, individuals in these groups had glycemic levels within the normal range (4% to 5.6%).^30^ These results reflect that the patients in groups 1 and 2 had poorly- and well-controlled T2DM, respectively. Since the glycemic levels were under control among patients in these groups, it is possible that these individuals were exposed to considerably less OS and accumulation of AGEs in the peri-implant tissues, thereby demonstrating low PISF CLs. Regarding the correlation of peri-implant probing depth with cortisol levels, regression analysis results showed that a statistically significant correlation existed between the aforementioned parameters only among patients in group 3. It has been proposed that periodontitis and peri-implantitis are linked with decreased whole salivary antioxidative activity and increased OS.^10,13,18,20,36,37^ Moreover, an increased peri-implant PD and simultaneous presence of periodontitis seems to be responsible for greater OS in the periodontal and peri-implant tissues. This factor may have contributed towards a statistically significant correlation between PISF CL and peri-implant PS. In contrast, despite the fact that patients in group 1 displayed probing depths comparable to those of patients in group 3, no statistically significant correlation was established between peri-implant PD and PISF CL. A definitive explanation for this is challenging; however, the authors of the present study perceive that the state of persistent hyperglycemia among patients in group 1 was the main contributor to elevated PISF CLs, wherease the contribution of peri-implant PD was less important. In further studies, it may be worthwhile to assess levels of AGEs and CL in the PISF of T2DM patients with peri-implantitis, which may demonstrate a statistically significant correlation.

A thought-provoking observation was that although the duration of type-2 diabetes was similar between patients in groups 1 and 2 (approximately 10 years), the clinicoradiographic status was statistically significantly poorer among patients in group 1 compared with group 2. One logical reason for this is that a marked difference existed in the HbA1c levels between patients in groups 1 and 2 with glycemic levels, being statistically significantly higher in the former. It is well documented that chronic hyperglycemia induces and promotes a state of OS in tissues, including those of the periodontium.^1^ Moreover, hyperglycemia is also linked with increased formation and accumulation of AGEs in periodontal and systemic tissues.^21,22,25^ These factors expose T2DM patients to an increased risk of periodontitis and peri-implantitis compared with systemically healthy individuals.^26,32^ The present authors support the finding of previous clinical investigations, that patients with poorly-managed T2DM had a worse peri-implant clinicoradiographic status than did systemically healthy controls.^5,6,35^ Simultaneously, there is abundant published clinical evidence in the indexed literature confirming that under optimal glycemic control, dental implants can osseointegrate and remain functional as well as esthetically stable in diabetic patients in a manner similar to non-diabetic individuals.^29^ In a systematic review, Naujokat et al^29^ stated that under optimal glycemic control, implant therapy is a predictable and safe procedure with a complication rate similar to that of non-diabetic individuals. The present study confirms the results reported by Naujokat et al.^29^

Psychological conditions such as anxiety and depression are often manifested in patients with diabetes.^8,9,28^ Moreover, increased CLs have been reported in the saliva of patients with depression and anxiety.^11,16,34^ In the present study, none of the patients reported having been diagnosed and/or being treated for psychological conditions. It is therefore perceived that the PISF CLs are statistically significantly higher and peri-implant clinicoradiographic inflammatory parameters are poorer among T2DM patients diagnosed with psychological conditions compared with diabetic patients without psychological disorders.

One limitation of the present study is that microbiological investigations were not performed. Moreover, patients using tobacco products were not included. Since some microbes, such as red-complex bacteria, and tobacco smoking are linked with the etiopathogenesis of peri-implant diseases,^3,31^ these parameters may be correlated with the expression of high concentrations of cortisol in these individuals. Further studies are needed to assess these hypotheses.

## Conclusion

Cortisol levels in PISF are higher in T2DM and healthy patients with peri-implantitis than individuals with without peri-implantitis. Hyperglycemia did not influence peri-implant clinicoradiographic parameters or CL in the present patient population.
